# Mild Depressive Symptoms in Airline Pilots Associated With Impaired Executive Functions

**DOI:** 10.7759/cureus.41616

**Published:** 2023-07-10

**Authors:** Piercarlo Minoretti, Andrés S Santiago Sáez, Ángel F García Martín, Miryam Liaño Riera, Manuel Gómez Serrano, Enzo Emanuele

**Affiliations:** 1 General Direction, Studio Minoretti, Oggiono, ITA; 2 Legal Medicine, Psychiatry, and Pathology, Complutense University of Madrid, Madrid, ESP; 3 Occupational Health, 2E Science, Robbio, ITA

**Keywords:** mental health, psychological welfare, executive fuctions, depressive symptoms, airline pilots

## Abstract

Introduction: While significant efforts have been made to understand the influence of depression-related impairments on executive functioning within the general population, the specific impact on airline pilots remains largely unexplored. Considering the crucial role that cognitive abilities play in the realm of aircraft piloting, it is imperative to probe into the potential repercussions of depressive symptoms on executive functions (EFs) among this professional cohort.

Objectives: This study aims to explore the associations between depressive symptoms and EFs in a convenience sample of airline pilots.

Methods: Male airline pilots (n = 100) underwent the Beck Depression Inventory II (BDI-II) to gauge both the presence and intensity of depressive symptoms. The Stroop Color and Word Test (SCWT), the Digit Span Task (DST), and the Wisconsin Card Sorting Test (WCST) were used to assess EFs.

Results: Of the entire sample of pilots, 88% (n = 88) demonstrated minimal depressive symptoms with a BDI-II score ranging from 0 to 13. The remaining 12% (n = 12) exhibited mild depression, with scores between 14 and 19. Pilots suffering from mild depression demonstrated prolonged color and word times and a higher time interference (TI) score on the SCWT. Moreover, these individuals exhibited lower scores on the DST across both the forward digit span (FDS) and backward digit span (BDS) subtests. Finally, the presence of mild depression correlated with an increased number of total errors, encompassing both perseverative and non-perseverative errors, in the WCST. After adjusting for potential confounding variables, we found an independent association between BDI-II scores and total errors in the WCST.

Conclusion: Our research points to substantial differences in EFs between airline pilots demonstrating mild depression and those exhibiting minimal depressive symptoms. This information can catalyze heightened consciousness about the psychological welfare of pilots.

## Introduction

Airline pilots routinely grapple with substantial stress levels, not only stemming from their profession’s responsibility but also from factors like disrupted circadian rhythms, diminished sleep quality, intense workload, and personal challenges [1,2]. Compounding these stressors can predispose pilots to mental health conditions, particularly depression [3]. Recent studies underscore that the prevalence of depression among airline pilots fluctuates between 1.9% and 12.6%, a rate that contrasts with the 7.2% to 12.9% observed in the general population [4]. This disparity in depression rates between pilots and the broader population indicates a potentially overlooked incidence of this affective disorder within the aviation community [4]. In a recent investigation, Becerril-Villanueva et al. [5] examined 79 airline pilots, 11 healthy volunteers, and 39 patients diagnosed with major depressive disorder (MDD). The study did not identify any airline pilots who met the diagnostic criteria for depression. However, it did uncover that both pilots and patients suffering from MDD displayed elevated mRNA levels of the serotonin transporter (SERT) and p11, two well-recognized biomarkers associated with stress and depression, when compared with healthy volunteers [5]. The authors concluded that airline pilots and depressed patients have similar alterations in their SERT and p11 expression levels, distinguishing them from control subjects and suggesting an enhanced vulnerability to depressive symptoms in this professional category [5].

Over time, the nature of aircraft piloting has evolved from primarily a physical and sensory-based activity to a cognitive role where the pilot functions as a supervisory system manager and decision-maker [6]. This evolution has underscored the importance of cognition, especially executive functions (EFs), as a significant predictor of flight performance [7,8]. EFs represent a diverse range of interconnected yet distinct advanced cognitive skills, essential for initiating goal-oriented actions and planning for future outcomes [9]. This multifaceted construct comprises three primary elements: cognitive flexibility, which enables the adjustment of thoughts to shifting demands; inhibitory control, which allows the suppression of impulsive responses; and working memory, a feature that facilitates the temporary storage and manipulation of information [10].

While significant efforts have been made to understand the effects of depression-related impairments on executive functioning within the general population [11-13], the specific impact on airline pilots remains largely unexplored. Given the vital importance of cognitive abilities in piloting [7,8], it becomes essential to delve into the potential impact of depressive symptoms on EFs in this professional group. As global air travel demand continues to grow, a more nuanced understanding of this relationship could pave the way for enhanced neuropsychological diagnostics within the aviation industry. Such advancements could also bolster flight performance and safety on a larger scale. Consequently, this study aims to explore the associations between depressive symptoms and EFs in a convenience sample of airline pilots.

## Materials and methods

Participants

During their routine occupational health visits at outpatient clinics (Studio Minoretti, Oggiono, Italy), 100 actively working male airline pilots of Caucasian descent voluntarily signed up for the study. An occupational health physician was responsible for sharing the invitation to participate. Due to the limited number of female pilots, they were not included in the study. The study excluded individuals with a known history of neurological, psychiatric, inflammatory, autoimmune, or infectious diseases. Those who had cancer or had taken medications in the past 90 days were also excluded. None of the participants were taking dietary supplements, and they all appeared to be in good physical health. Each participant provided their written consent before the study began. The research followed the ethical guidelines of the Declaration of Helsinki and received approval from the local ethics committee (reference number: 2021/04E).

Depressive symptoms

Each participant underwent evaluation based on the Beck Depression Inventory II (BDI-II). This self-reporting instrument, which consists of 21 items, is designed to gauge both the presence and intensity of depressive symptoms [14]. The scores derived from this tool denote varying levels of depressive severity. Scores 0-13 represent minimal depressive symptoms, 14-19 suggest mild depression, 20-28 indicate moderate depression, and 29-63 indicate severe depression [14].

Stroop Color and Word Test

The Stroop Color and Word Test (SCWT) is a neuropsychological instrument used to measure cognitive processing speed, selective attention, cognitive flexibility, and inhibitory control [15]. This test challenges participants to read color names that are printed in contrasting colors, such as the word “blue” displayed in red ink. The speed and accuracy of their responses serve as significant indicators of their ability to suppress instinctive reactions and engage in more complex, unfamiliar tasks. The SCWT is designed around three unique cards. The first, known as the color card, contains 24 dots, each depicted in one of four distinct colors: red, yellow, blue, or green. The second, referred to as the word card, features 24 words, each displayed in the same quartet of colors. The third and final card is the color-word card, which showcases 24 color-words printed in contrasting colors to their meaning, such as the word “blue” appearing in red ink. Participants are tasked with identifying the colors of the dots or words on each card as quickly and accurately as they can. The time taken to read each card, the color card, word card, and color-word card, is represented by the symbols CT, WT, and CWT, respectively. The time interference (TI) score, a critical measure in the SCWT, is then derived using the formula: TI = CWT - (WT + CT) [15].

Digital Span Task

The digit span task (DST) is a critical tool that assesses verbal short-term and working memory through two subtests: forward digit span (FDS) and backward digit span (BDS) [16]. Each subtest comprises two sets of digit sequences. During the test, the examiner reads aloud numbers from the first sequence at a steady pace of one number per second. The participant is then required to replicate the FDS sequence in the original order and the BDS sequence in reverse order. Upon a correct response, the task advances to the next sequence. However, if an error occurs, the same sequence from the second set is presented. The task ceases after two successive errors on sequences of the same length. The participant’s score is calculated as the total number of correctly recalled sequences prior to the occurrence of two consecutive errors. As the number of digits in the sequences increases, both the FDS and BDS subtests become progressively more challenging [16].

Wisconsin Card Sorting Test

The Wisconsin Card Sorting Test (WCST) is a neuropsychological test designed to assess abstract reasoning abilities and the ability to change cognitive strategies in response to changing environmental contingencies [17]. It measures cognitive flexibility, problem-solving, and strategizing skills, particularly within the realm of EFs. The WCST utilized in this research employed a computerized 128-card version. This version incorporates 128 response cards and four stimulus cards, each featuring diversified shapes, colors, and numerals. The primary task asked of participants was to align the geometrical figures on the response cards to the corresponding ones on the stimulus cards, guided by feedback provided after each response. Following a sequence of 10 correct responses, the sorting rule would subsequently alter. Key statistical indicators employed within this study included the percentage of perseverative errors, non-perseverative errors, and the total number of errors [17].

Statistics

Given that no airline pilot in our study scored 20 or above on the BDI-II, we categorized our sample into two groups based on recognized threshold scores. These included a 0-13 group, indicative of minimal depressive symptoms, and a 14-19 group, representing mild depression. We expressed categorical variables as counts and percent frequency and used the chi-square test for comparing these variables between the two groups. Continuous variables were presented as means ± standard deviations and were compared using unpaired Student’s t-tests. Through multivariable stepwise linear regression analyses, we identified independent associations between BDI-II scores and EF test results after adjustment for age, BMI, and education levels. We conducted all analyses using SPSS Statistics version 20.0 (IBM Corp. Released 2011. IBM SPSS Statistics for Windows, Version 20.0. Armonk, NY: IBM Corp.), with statistical significance being determined by a two-tailed p-value less than 0.05.

## Results

Of the entire sample of pilots (n = 100), 88% (n = 88) demonstrated minimal depressive symptoms with a BDI-II score ranging from 0 to 13. The remaining 12% (n = 12) exhibited mild depression, with scores between 14 and 19. Table 1 illustrates the general characteristics of these two groups. A comparative analysis revealed no significant intergroup differences in terms of age, BMI, and educational levels.

**Table 1:** General characteristics of airline pilots categorized according to the intensity of depressive symptoms BMI: body mass index; BDI-II: Beck Depression Inventory II; ns: not significant

	Pilots with mild depression (n = 12)	Pilots with minimal depressive symptoms (n = 88)	P
Age, years	40 ± 5	39 ± 4	ns
Male sex, n (%)	12 (100)	88 (100)	ns
BMI, kg/m^2^	24 ± 2	23 ± 3	ns
Education, years	17 ± 2	17 ± 3	ns
BDI-II score	16 ± 2	5 ± 3	<0.001

EFs according to the presence and severity of depressive symptoms are summarized in Table 2.

**Table 2:** EF results of airline pilots categorized according to the intensity of depressive symptoms SCWT: Stroop Color and Word Test; CT: time to read the color card; WT: time to read the word card; CWT: time to read the color-word card; TI: time interference; DST: digit span task; FDS: forward digit span; BDS: backward digit span; WCST: Wisconsin Card Sorting Test; ns: not significant

	Pilots with mild depression (n = 12)	Pilots with minimal depressive symptoms (n = 88)	P
SCWT			
CT (sec)	16 ± 5	17 ± 4	ns
WT (sec)	16 ± 7	16 ± 8	ns
CWT (sec)	26 ± 8	22 ± 10	<0.05
TI	9 ± 3	6 ± 2	<0.05
DST			
FDS	8 ± 1	10 ± 2	<0.05
BDS	5 ± 1	7 ± 2	<0.05
WCST			
Total errors	35 ± 5	27 ± 7	<0.001
Percent perseverative errors	16 ± 3	13 ± 5	<0.05
Percent non-perseverative errors	12 ± 2	10 ± 2	<0.05

In the SCWT, pilots with mild depression showed a significantly elongated CWT duration (26 ± 8 sec) compared to those with minimal depressive symptoms (22 ± 10 sec, p < 0.05). They also had a higher TI score (9 ± 3), again statistically significant compared to the minimal depression group (6 ± 2, p < 0.05), hinting toward a greater Stroop interference effect. Nevertheless, when comparing both the CT and WT durations, no significant intergroup differences were observed. In the DST, pilots with mild depression were able to repeat significantly fewer series of digits in both FDS and BDS subtests. In the FDS subtest, their average was 8 ± 1 compared to the 10 ± 2 average of those with minimal symptoms (p < 0.05). Similarly, in the BDS subtest, the average was 5 ± 1 for pilots with mild depression versus 7 ± 2 for those with minimal symptoms (p < 0.05). In the WCST, pilots with mild depression displayed a greater number of total errors, averaging 35 ± 5, compared to their counterparts with minimal depressive symptoms who averaged 27 ± 7 (p < 0.001). The same pattern was observed in the percentage of perseverative errors, where pilots with mild depression scored 16 ± 3, which was higher than the 13 ± 5 scored by the pilots with minimal depressive symptoms (p < 0.05). Similarly, in the case of non-perseverative errors, the pilots with mild depression had a higher percentage (12 ± 2) versus those with minimal symptoms (10 ± 2, p < 0.05). In the multivariable stepwise linear regression analysis conducted to examine associations between BDI-II scores and EFs, the sole independent association identified was with total errors on the WCST (beta = 0.58, p < 0.05).

## Discussion

Our current research reveals a 12% occurrence of mild depression among airline pilots, a statistic that aligns with the previously reported 1.9−12.6% range by Pasha and Stokes in their literature review [4]. This is, to our knowledge, the first study to highlight significant differences in EFs between airline pilots with mild depression compared to those with minimal depressive symptoms. Specifically, we arrived at four principal conclusions. Firstly, pilots suffering from mild depression demonstrated prolonged CWT times and higher TI scores on the SCWT. Secondly, these individuals exhibited lower scores on the DST across both FDS and BDS subtests. Thirdly, the presence of mild depression correlated with an increased number of total errors, encompassing both perseverative and non-perseverative errors, in the WCST. Lastly, after adjusting for potential confounding variables, we found an independent association between BDI-II scores and total errors in the WCST.

The SCWT serves as an effective tool for evaluating cognitive flexibility and the capacity to manage cognitive interference [15]. Specifically, the time taken to complete the CWT is indicative of an individual’s efficiency in recognizing and labeling colors and words [15]. In the case of pilots exhibiting mild depression, a longer CWT completion time compared to those with minimal depressive symptoms suggests a reduced processing speed. Moreover, higher TI scores on the SCWT imply an increased level of cognitive interference [15]. This indicates that airline pilots with mild depression may find it more challenging to inhibit cognitive interference, thereby struggling with the effective management of conflicting information. The DST is a cognitive test that measures working memory and attention span. The FDS subtest requires the individual to repeat a series of numbers in the same order they were presented, while the BDS subtest requires the individual to repeat the series of numbers in the reverse order [16]. Therefore, reduced performance on both these subtests in pilots with mild depression suggests difficulties with basic information processing and working memory, along with the ability to manipulate information. Finally, the study revealed that pilots with mild depression exhibited a higher error rate on the WCST, indicating potential difficulties in problem-solving, adaptive thinking, and decision-making abilities [17]. Moreover, mild depression in airline pilots correlated not just with a greater total error count on the WCST but also with both perseverative and non-perseverative errors. The notable association with non-perseverative errors potentially signifies an impairment in cognitive flexibility [17]. Intriguingly, the severity of depressive symptoms was independently associated with the total amount of errors. This suggests that as depressive symptoms intensify, the probability of committing errors on the WCST may escalate as well, irrespective of any other confounding factors.

These findings collectively illuminate the potentially harmful impact that depressive symptoms could exert on aviation, where optimal EFs are vital for maintaining both flying performance and safety [7,8]. For example, prior studies have observed a persistent pattern among pilots to continue landing despite challenging conditions, with poor crosswind landing decisions making up the majority of incidents [8,18]. Notably, working memory updating has been identified as a key factor in predicting optimal crosswind landing decisions, differentiating between pilots who make correct decisions and those who do not [8]. In addition, a strong correlation was observed between working memory span and aviation communication performance [19]. This correlation aligns with expectations, given that aviation communication heavily involves working memory. Pilots are indeed required to recall information from air traffic control, relay the details, and then adjust aircraft flying accordingly. Unfortunately, miscommunication or misinterpretation of messages from air traffic control, potentially due to impaired EFs, is a common source of errors [19]. Considering the harmful effects of depressive symptoms on EFs unveiled in our study, along with the clear safety implications, it becomes imperative to establish proactive mental health screenings and support services for pilots [20]. Addressing and treating depressive symptoms in this context could potentially enhance EFs and, consequently, improve job performance and safety outcomes.

Our study, primarily concentrated on occupational medicine, validates previous findings from the general population that depressive symptoms can cause deficiencies in EF domains. For instance, as far back as 1997, Lamelin et al. [21] highlighted a compromised performance on the SCWT amongst depressed individuals. This points to the presence of two separate attention deficit patterns in clinical depression: one group struggles with inhibiting distractions, while the other faces insufficiencies in processing resources. A classical study conducted by Merriam et al. [22] revealed that individuals suffering from depression exhibited notable impairments across various measures of the WCST when compared to healthy controls. Moreover, the severity of depression was found to be positively correlated with the extent of these deficits [22]. Finally, a comprehensive meta-analysis of 22 studies revealed that individuals diagnosed with MDD exhibited significant impairments on the DST, in comparison to the performance of healthy participants [23].

Our study does have a few salient limitations that necessitate further comment. Firstly, the disparity in the size of the two groups may have minimized the effect size in our study when comparing pilots with minimal depressive symptoms against those with mild depressive symptoms. Moreover, the lack of female participants, despite the sex distribution within our sample being representative of the wider demographic of airline pilots [5], may limit the applicability of our results to women specifically. Consequently, it is essential to conduct more extensive studies with a larger and more diverse cohort to verify and broaden our findings. Secondly, data on socioeconomic status and physical activity levels, which could potentially impact EFs, were not gathered as they fell outside the purview of routine occupational medicine visits. Hence, it is imperative for subsequent studies to investigate these and other factors that could serve as potential confounding variables in the association between depressive symptoms and EFs in airline pilots. Lastly, it is important to note that our research had a cross-sectional nature. As such, while we were able to discern associations, we could not definitively establish causation or make predictions.

## Conclusions

Despite these limitations, our research points to substantial differences in EFs between airline pilots demonstrating mild depression and those exhibiting minimal depressive symptoms. This information can catalyze heightened consciousness about the psychological welfare of pilots. By doing so, we can bolster the safety and efficiency of aviation operations, thus emphasizing the critical need to address mental health in this high-stakes profession.
